# All Saints Convalescent Hospital, Eastbourne

**Published:** 1894-12-08

**Authors:** 


### The Institutional Workshop.

### WITHIN THE HOSPITALS.

By Our Special Commissioner.

### ALL SAINTS' CONVALESCENT HOSPITAL, EASTBOURNE.

The All Saints' Convalescent Hospital and Children's Hospital was established in 1864, and is one of the oldest and largest institutions of the kind in the world. It contains 500 beds, 100 of which are for children, who occupy a separate building, replete with everything calculated to promote their recovery and conduce to their comfort and happiness. It is, we believe, true that the whole of the administration and much of the work is done gratuitously, a fact which might prepare one to find that relatively the management was somewhat slack, if not inefficient. A careful inspection of the institution, which, by the courtesy of the sister in charge, we were able to make on a day when the buildings are not open to visitors, convinces us, that the voluntary service rendered to so large an extent by those who devote their lives to tending the sick in this institution is of a high order and special value. It is not sufficiently realized by the public that a superior class of patients have been provided for by the addition of some private bed-rooms with a common sitting-room, upon the payment of 15s. a week per patient. Another feature of the institution of special value, is the provision of six beds for married women with their infants. This latter branch of the work is not as widely known as it ought to be, and we should like to hear that these beds are never vacant, because they afford an opportunity for district nurses and visitors to render immense service to the mothers in crowded cities, many of whom, owing to the conditions under which they habitually live, find Eastbourne a paradise upon earth. At the time the present buildings were erected they were far away from all houses, and enjoyed an uninterrupted view of the Downs on one side and of the sea on the other. The site is well selected and most attractive, but the building operations of late years have caused a number of houses to be erected in the immediate vicinity of the hospital, which has interfered with the view of the Downs.

#### The Convalescent Hospital.

The buildings devoted to the reception of adults were erected thirty years ago, at a time when the requirements of convalescent patients were not fully appreciated. They contain well proportioned dormitories, which are, probably, as perfect as good planning and hygienic conditions can make them. Some of the fittings are necessarily old, but taken as a whole the sleeping and lavatory accommodation upstairs in this building is distinctly good. Down stairs, however, there is an absence of light and ventilation, which it is difficult now to alter, and the day-room space, when the hospital is full, is so inadequate as to necessitate further provision in this direction without delay. Those who are contemplating the erection of a Convalescent Home should visit the Hospital at Eastbourne during the months when it is usually full, so that they may realise what to avoid in the way of inadequate day-room space for the patients. The kitchen is well situated, well conducted, and adequate. The dining halls for men and women, the class of food supplied, and the service all appear good and satisfactory. The laundry is distinct from the hospital proper, of adequate proportions, and we believe well managed also. There is a finely proportioned chapel, which has unfortunately at present little or no ventilation. This defect must make it anything but a pleasant place when it is crowded with worshippers, and as it might be well ventilated by the expenditure of a reasonable sum, we hope that early attention will be given to this necessary improvement.

#### The Children's Hospital.

The Children's Hospital is a much later building, which was erected as a memorial to the late mother foundress, Harriet Brownlow Byron, and was opened by their Royal Highnesses the Prince and Princess of Wales, accompanied by the Princesses Victoria and Maud of Wales, in 1891. It is in all respects better planned than the old building, though we fancy from what we saw and heard that it was erected rather too cheaply, and that in consequence repairs and alterations are likely to amount to a considerable sum from year to year. The day-rooms are bright, airy, well-proportioned, pleasant, and cheerful. They contain a great number of playthings and apparatus for the amusement of the children, and, like the refectory where the children take their meals, deserve to be described as excellent. It did the visitor's heart good to hear the merry laughter and see how happy and bright all the children were. Tears are, we fancy, seldom shed by the occupants of this hospital, where everything has been done to promote happiness and secure speedy recovery. The lavatories are specially good, and are more numerous than in the older building devoted to adults. If we might venture a criticism, it would be in the direction of suggesting that additional Tobin's tubes should be placed in the dormitories, which are liable to become close and stuffy, especially when the state of the weather necessitates the closing of the windows during the greater part of each twenty-four hours. As an illustration of the joys which little children are likely to experience in this home of rest, we may point to the fully-equipped ship, rigged and manned, which occupies a central position in one of the day-rooms. There are also shops of various kinds, which have been brought together in one large dolls' house, so as to represent in miniature a great co-operative store, where everything can be purchased. Here the children have an opportunity of seeing all sorts of things, as the contents of the shops are very realistic and have been most admirably managed and arranged. There is a dolls' hospital, too, where the builder has expended considerable ingenuity, close attention to details, and a complete knowledge of the work that is done in the various parts of a great hospital every day. We do not ever remember seeing anything more perfect of their kind than the ship, the stores, and the dolls' hospital. We should like to take some really wealthy people into these day-rooms, and show them not only the children and the accommodation provided for them, but these beautiful fittings and objects of interest and instruction, because we feel confident that once seen they would not easily be forgotten, and that everyone of them who had the means would cheerfully give of their abundance to support so excellent an institution as the Children's Hospital at Eastbourne. Of all the institutions of which her Majesty the Queen is a patron, we doubt if there is one which would more command her Majesty's admiration and sympathy than the children's hospital we have just described.

How to Help Convalescents.

For some reason, which it is hard to explain, convalescent patients are not sent in any number to Eastbourne during the late autumn and winter months. Yet Eastbourne is a popular place of resort for the well-to-do during this period of the year, and, as we stated in a recent article, there are few places on the sea coast which are better suited for a winter residence, or where greater advantages can be reaped by an invalid who requires really bracing air and invigorating freshness. Why is it, then, that more patients are not sent to Eastbourne in the winter, as they undoubtedly ought to be? We commend this question to the consideration of hospital authorities, and to all who are in the habit of sending patients there during the summer months, when they often find it difficult to obtain admission for their protegés, who would, in the majority of cases, benefit to as great, if not even to a greater extent, if sent during the autumn and winter months when, under present conditions, there is an abundance of room. As with the adults so it is with the children. Some children, especially boys, would be heartily welcome at the Convalescent Hospital during the months from October to April inclusive. There is every provision for bad weather, as the children are provided with a covered open playground, in addition to the gardens and grounds. We hope that, as one result of our inspection, many more adults and children will be sent to Eastbourne during the months in question from this date. Fashion dominates the medical as well as other worlds to a very large extent, but we think it is altogether ridiculous to send a number of sick people into a convalescent home during a few months only of each year, when the whole institution is crowded, if not overcrowded, and less rather than greater benefit is likely to result to the patients. From October to April there would be many advantages gained by the patients from which they would be debarred at a time when all the beds were occupied. As the building exists, and the staff is ready to receive patients, we hope that these may be sent in increasing numbers every year during the months in question, when no doubt a proportionate increase will be made in the revenues of this great institution, which has done so much to restore the bread-winner to health, and so to make him or her a better citizen and parent.

Mothers and Babies.

One more thing we should like to see, and that is more mothers with their babies applying for admission to the six beds in two rooms which are devoted to the reception of this class of case. Here, at least, there can be no doubt that if the vacancies were widely known and understood, applicants would be forthcoming in much larger numbers than the accommodation could provide for. It was a genuine pleasure to inspect this institution and to see the devotion to duty and the high class of work which is being done by the sisters and nurses under their charge. It is further gratifying to notice that the whole administration is under the control of a comparatively small committee, the members of which rightly regard it as a privilege to spend and be spent in promoting the best interests of an institution which, as we have stated, does immense good to thousands of our poorer population every year.

